# Unexpected Instability of Family of Repeats (FR), the Critical *cis*-Acting Sequence Required for EBV Latent Infection, in EBV-BAC Systems

**DOI:** 10.1371/journal.pone.0027758

**Published:** 2011-11-16

**Authors:** Teru Kanda, Sachiko Shibata, Satoru Saito, Takayuki Murata, Hiroki Isomura, Hironori Yoshiyama, Kenzo Takada, Tatsuya Tsurumi

**Affiliations:** 1 Division of Virology, Aichi Cancer Center Research Institute, Kanokoden, Chikusa-ku, Nagoya, Japan; 2 Research Center for Infection-associated Cancer, Institute for Genetic Medicine, Hokkaido University, Kita-ku, Sapporo, Japan; 3 Department of Tumor Virology, Institute for Genetic Medicine, Hokkaido University, Kita-ku, Sapporo, Japan; Louisiana State University Health Sciences Center, United States of America

## Abstract

A group of repetitive sequences, known as the Family of Repeats (FR), is a critical *cis*-acting sequence required for EBV latent infection. The FR sequences are heterogeneous among EBV strains, and they are sometimes subject to partial deletion when subcloned in *E. coli*-based cloning vectors. However, the FR stability in EBV-BAC (bacterial artificial chromosome) system has never been investigated. We found that the full length FR of the Akata strain EBV was not stably maintained in a BAC vector. By contrast, newly obtained BAC clones of the B95-8 strain of EBV stably maintained the full length FR during recombinant virus production and B-cell transformation. Investigation of primary DNA sequences of Akata–derived EBV-BAC clones indicates that the FR instability is most likely due to a putative secondary structure of the FR region. We conclude that the FR instability in EBV-BAC clones can be a pitfall in *E. coli*-mediated EBV genetics.

## Introduction

Epstein-Barr Virus (EBV), a member of the gamma herpesvirus family, is a human tumor virus that is associated with various neoplastic diseases, such as Burkitt's lymphoma, nasopharyngeal carcinoma, Hodgkin's lymphoma, and opportunistic lymphomas in immunosuppressed transplantation recipients [1]. The EBV genome is a linear, double-stranded, 175-kb genome, in which more than 80 viral genes are encoded [2], [3]. Like other members of the herpesvirus family, the EBV genome contains multiple repetitive sequences. For example, the genome has four major internally located repeats (Internal Repeats, IR1 through 4), as well as terminally located repeats (Terminal Repeats, TR) on both ends of its linear genome [3]. When EBV infects cells, the genome becomes an episome (double-stranded circular genome) with a characteristic number of TRs [4]. In addition, the EBV genomes have another group of repetitive sequences, designated as Family of Repeats (FR) [2], [5], [6], which are located within the *oriP* region of the genome [7]. These repetitive sequences (IRs, TR, and FR) consist of unique sequences within loci made up of varied numbers of repeats.

The FR consists of multiple copies of 30-bp repeat units [6]. Each repeat unit contains a binding site for viral protein EBNA1, which plays important roles in EBV latent infection [6]. Several critical roles have been assigned to the FR for the maintenance of EBV latent infection [8], [9]. The binding of the EBNA1 protein to the FR sequence enables EBV genomes to be maintained as episomes, and it enhances the activity of viral latent promoters, which drive the expression of viral transforming gene products [10]. Although the copy number of the FR repeats varies substantially in different EBV strains, the length of the FR within each EBV strain is stably maintained throughout long-term passage [5], [11]. The B95-8 strain of EBV [12] contains 29 copies of FR repeats [5], [11], each copy 30-bp long, although it was formerly thought that this strain contained only 20 copies [2], [8], [9]. On the other hand, the Burkitt's lymphoma-derived Akata strain of EBV [13] contains 32 copies of the FR sequence [11].

It has been some time since bacterial artificial chromosome (BAC) systems were introduced into the field of EBV virology [14]. The EBV-BAC system enables precise and rapid engineering of the large EBV genome by means of very efficient homologous recombination in *E. coli* [15]. As with the B95-8 strain of EBV [14], the genome of the Akata strain of EBV [16] has been cloned into BAC vectors. The EBV-BAC system, which utilizes the B95-8 strain, has been extensively used, since this system represents the sole system that enables the production of BAC-derived pure recombinant viruses. A drawback of the BAC system is the possible instability of viral repetitive sequences upon propagation of viral genomes in *E. coli*. To our knowledge, the integrity of repetitive sequences of EBV-BACs has never been verified in detail to date. We recently found that maintenance of the full-length FR is critical to the efficient transformation of B-lymphocytes by EBV [11]. It is therefore important to characterize whether, and to what extent, EBV-BAC clones keep these repetitive sequences intact.

This study aims to clarify whether the FR stability in BAC vectors differs between EBV strains. We provide evidence that the FR of the Akata strain EBV could not be maintained as its original size in a BAC vector, as far as DH10B *E. coli* is used as a bacterial host. By contrast, the full length FR of the B95-8 strain EBV was stably maintained in a BAC vector, and during recombinant virus production and B cell transformation as well. Investigation of primary DNA sequence of the Akata strain FR provides clues to how it becomes unstable in *E. coli*-based cloning vectors.

## Results

### The FR sequence of the Akata-derived BAC clone is unstable in *E. coli*

**Figure 1:**
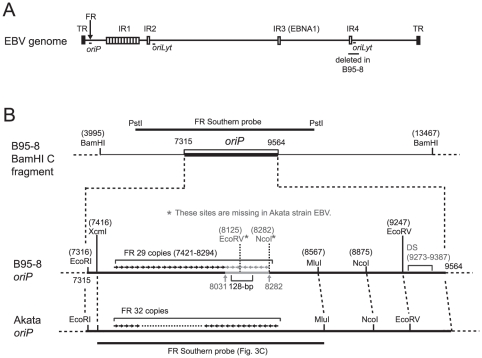
Schematic overview of EBV repetitive sequences and detailed maps of *oriP*. (A) Schematic representation of the various repetitive sequences within the EBV genome. Internal repeats (IR) 1 to 4 are indicated [3]. The *oriP* region (spanning the FR region), the *oriLyt* regions [18], and the region deleted in the B95-8-strain EBV (spanning the region of IR4 and the right *oriLyt*) are also indicated. (B) Restriction enzyme maps of the B95-8 and the Akata *oriP* regions. The 252-bp sequence that is missing in the B95-8 sequence (V01555) is indicated in gray within the B95-8 *oriP*. The position of the 128-bp palindromic sequence is also indicated. The numbering of the base pairs corresponds to the B95-8 sequence (V01555), except for the addition of 252 bp in the downstream region of the FR.

Various repetitive sequences are scattered throughout the EBV genome, and the FR sequence is in the *oriP* region (Fig. 1A). The restriction enzyme map of *oriP* regions of the B95-8 strain EBV and Akata strain EBV are schematically drawn in Fig. 1B. The FR sequence of the B95-8 strain of EBV has a 128-bp palindromic sequence in its 3′ end [5], and the 252-bp sequence containing this palindromic sequence tends to be deleted when subcloned in *E. coli*-based plasmid vectors (Fig. 1B).

**Figure 2:**
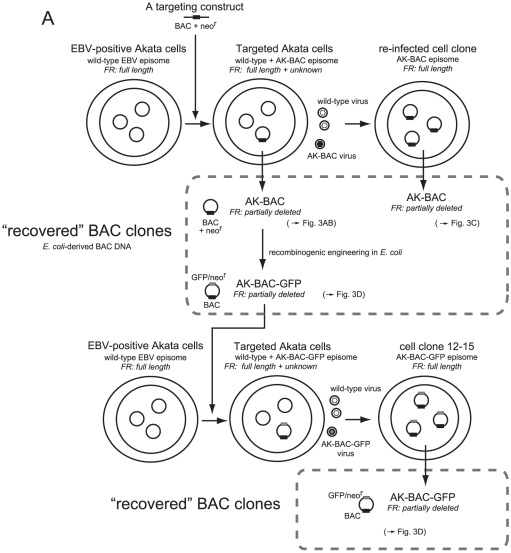
A lineage map of BAC clones of the Akata strain EBV. Latently-infected EBV episomes are schematically drawn as small circles in cell nuclei. Transgenes (BAC: BAC vector sequence, neo^r^: neomycin resistance gene, and GFP) are indicated as small boxes (either black or gray) on the circles. The estimated FR lengths of various BAC clones are also indicated.

**Figure 3:**
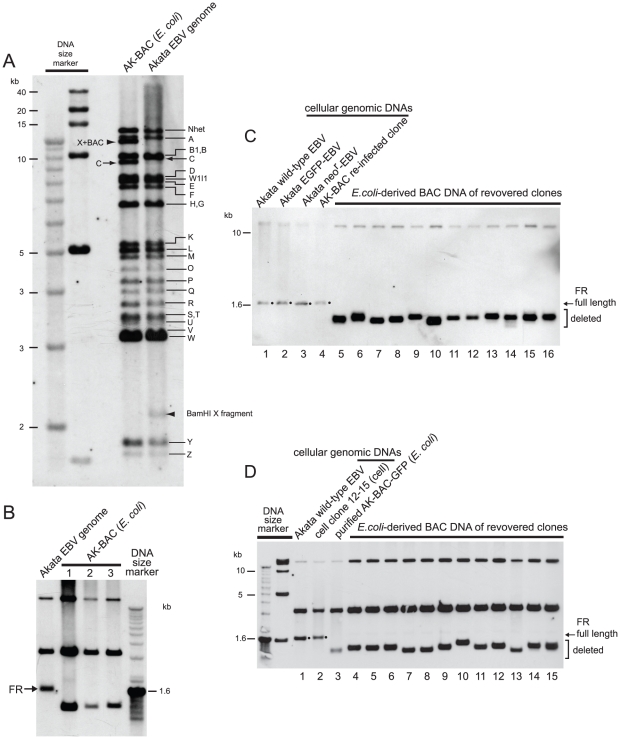
The FR instability of the Akata strain EBV-BAC clones. (A) *E. coli*-purified AK-BAC DNA and the genomic DNA of Akata cells harboring wild-type episomes were digested with BamHI and subjected to Southern blot analysis. A pool of BamHI-digested restriction fragments of AK-BAC was used as a probe. The BamHI-digested bands of Akata EBV genome are indicated by alphabets, and the BamHI C bands spanning the FR region are indicated by arrows. The altered bands due to the insertion of transgenes are indicated by arrowheads. (B) The genomic DNA of Akata cells and the *E. coli*-purified DNAs of three independent AK-BAC clones were double-digested with EcoRI and NcoI and analyzed by Southern blot analysis using the DNA fragment spanning the FR region as a probe (Fig. 1B). (C) The genomic DNAs of Akata cells and two derivative cells lines (lanes 1 through 3), the genomic DNA of re-infected cell clone (Fig. 2A), and DNAs of 12 independent AK-BAC clones recovered from the re-infected cell clone (lanes 5 through 16) were digested with EcoRI and NcoI and subjected to Southern blot analysis using an FR specific probe (XcmI-MluI fragment of B95-8 *oriP* in Fig. 1B). The bands representing the full-length FR are indicated by dots. (D) The genomic DNA of Akata cells harboring wild-type EBV (lane 1) and AK-BAC-GFP (lane 2), bacterially-prepared AK-BAC-GFP DNA (lane 3), and DNAs of 12 independent AK-BAC-GFP clones recovered from latently infected cells (lanes 4 to 15) were subjected to Southern blot analysis as in (B).

We previously reported the cloning of the entire EBV genome of Akata strain into a BAC vector [16]. An Akata-derived BAC clone, designated as AK-BAC, was obtained by transforming DH10B *E. coli* with genomic DNA of EBV-positive Akata cells harboring both wild-type episomes and targeted episomes [16] (Fig. 2A). We developed a novel experimental strategy to compare the restriction enzyme-digested fragments of AK-BAC and those of wild-type Akata strain EBV genome from which it is derived. In this experimental strategy, a pool of BamHI-digested restriction fragments of AK-BAC was labeled and used as a probe for Southern blot analysis. We found that the BamHI C fragment of AK-BAC was slightly shorter than that of wild-type Akata EBV genomes (Fig. 3A), which was overlooked by the restriction enzyme mapping in our study [16]. All the other restriction fragments were found to be intact sizes, except for those that had been generated by the insertion of transgenes (a BAC vector and a neomycin resistance gene).

Since the FR sequence was within the BamHI C fragment of EBV genome, we used Southern hybridization to verify the FR sizes of AK-BAC clones. The EcoRI-NcoI fragment of wild-type EBV genome, spanning the FR region, was larger than 1.6 kb marker DNA (Fig. 3B). By contrast, three of the examined AK-BAC clones had the FR sequences with approximately 1.2 kb (Fig. 3B), which was approximately 400-bp smaller than the full length FR of the Akata strain EBV. The FR sizes of three BAC clones appeared to be very similar, but not necessarily identical, to each other.

The above result implies that FR deletion occurred during AK-BAC episomes being transferred to *E. coli* or during being propagated in *E. coli*. However, a possibility remained that peculiar episomes with partially deleted FR were targeted in Akata cells, and that such peculiar episomes were recovered as AK-BAC clones. In order to rule out such a possibility, we extended the study to various derivative cell lines harboring AK-BAC. A lineage map of various cell clones is schematically illustrated in Fig. 2A. First, a cell clone, designated as “a re-infected cell clone”, was established by infecting EBV-negative Akata cells with a mixture virus consisting of wild-type virus and AK-BAC virus, followed by G418 selection. A PCR analysis of the genomic DNA of the “re-infected cell clone” demonstrated that the cell clone harbored only AK-BAC episomes and was free of wild-type episome (Fig. S1). We found that the FR size of AK-BAC episomes in the re-infected cell clone was identical to wild-type Akata strain EBV episomes (Fig. 3C, lane 4). This observation fits well with our previous data that the FR length is stably maintained during progeny virus production and infection to other cells [11]. The episomal DNAs were then prepared from the re-infected cell clone, and were electroporated into DH10B competent cells to recover BAC clones from the cells. Chrolamphenicol-resistant colonies were inoculated into bacterial culture medium, propagated over night at 37°C, and BAC clone DNAs were prepared by minipreparation. The FR sizes of 12 independent “recovered” clones were then examined by Southern blot analysis. We found that the FR sizes of the *E.coli*-derived BAC DNA were heterogeneous (Fig. 3C, lanes 5 through 16), and that all of them were smaller than those in wild-type episomes in latently infected cells (Fig. 3C, lane 1). Thus, we conclude that the FR sequences of AK-BAC episomes were full length in the re-infected cell clone, but they were partially deleted during AK-BAC episomes being transferred to *E. coli* or during being propagated over night in *E. coli*.

### Generality of the FR instability of Akata-derived EBV-BAC clones

In order to clarify whether the FR instability is a general problem of Akata-derived BAC clone, we subjected another BAC clone, AK-BAC-GFP (clone 12–15), for the same analysis as described above. AK-BAC-GFP, an AK-BAC derivative, was generated by putting GFP and neomycin resistance transgenes into AK-BAC via recombinogenic engineering in *E. coli* [16] (Fig. 2A). The FR sequences of *E. coli-*purified AK-BAC-GFP DNA was also partially deleted (Fig. 3D, lane 3), which was expected as its parental clone AK-BAC had the deleted FR (Fig. 3B). As described in detail in our previous study [16], the cell clone 12–15, harboring AK-BAC-GFP episomes, was established by introducing the bacterially prepared AK-BAC-GFP DNA (with partially deleted FR) into EBV-positive Akata cells (harboring wild-type EBV), and by subsequently using the mixed-population virus (wild-type virus and AK-BAC-GFP virus) to infect EBV-negative Akata cells (Fig. 2A). Intriguingly, the FR sizes of AK-BAC-GFP episomes in latently infected cells were identical to those of wild-type EBV episomes (Fig. 3D; compare lanes 1 and 2). Since only wild-type EBV episomes can provide AK-BAC-GFP with the full length FR in this experimental setting, this observation implies that the shortened FR of bacterially prepared AK-BAC-GFP had been repaired via homologous recombination with wild-type episomes during the course of establishing the cell clone 12–15.

EBV-BAC clones were then recovered from the cell clone 12–15 (harboring AK-BAC-GFP episomes) as described above. Again, Southern blot analysis revealed that the FR sizes of the *E.coli*-derived BAC DNA were heterogeneous (Fig. 3D, lanes 4 through 15), and that all of them were smaller than those in AK-BAC-GFP episomes in latently infected cells (Fig. 3D, lane 2). More BAC clones were recovered from the same cells, but none of them retained the full-length FR (Fig. S2).

In summary, EBV-BAC episomes in re-infected clone and the cell clone 12–15 (Fig. 2A) both retained the full length FR. However, none of the bacterially prepared EBV-BAC clones that were recovered from these cell clones retained the full length FR, and all harbored partially deleted FR. The results strongly argue against the possibility that the FR instability noted with the AK-BAC being just a specific clone. Therefore, we conclude that this is a general problem of trying to work with the Akata strain in BACs.

### Cloning of B95-8-strain EBV with the full-length FR

Our recent study demonstrated that the full-length FR from the B95-8 strain of EBV can be used to replace the partially deleted FR from AK-BAC [11]. Thus, we envisioned that it should be feasible to clone the entire genome of B95-8-strain EBV while keeping its FR sequence intact. The EBV-BAC clone containing the entire genome of B95-8-strain EBV has been widely used [14], but the FR size of the BAC clone has never been examined. Thus, we set out to clone the genome of B95-8-strain EBV into a BAC vector while paying special attention to the integrity of the repetitive sequences, including the FR sequence.

**Figure 4:**
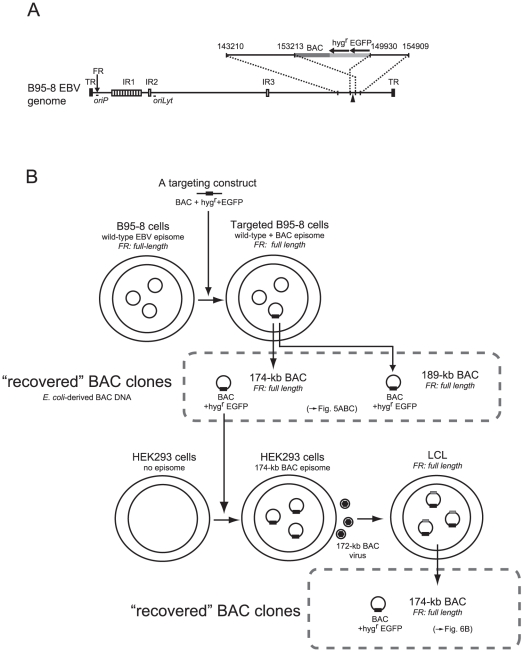
A lineage map of newly obtained BAC clones of the B95-8 strain EBV. (A) A schematic representation of the B95-8-strain EBV genome and the insertion locus of transgenes. The breakpoint of the B95-8 deletion is indicated by an arrowhead. The numbering of the base pairs, indicated for the right and left homology regions, corresponds to the B95-8 sequence (V01555). Transgenes (BAC: BAC vector sequence, hyg^r^: hygromycin resistance gene, and EGFP gene) are indicated. (B) A lineage map of BAC clones derived from the B95-8 strain EBV is shown. BAC clones were recovered from the targeted B95-8 cells, and then transferred back into HEK293 cells. LCLs were then established from the recombinant viruses obtained from BAC-transduced HEK293 cells.

**Figure 5:**
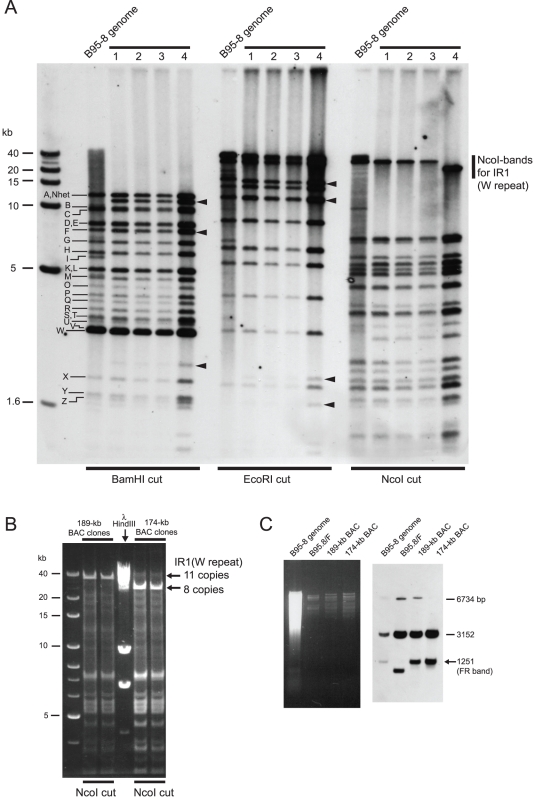
Stability of repetitive sequences in the newly-obtained EBV-BAC clones. (A) The genomic DNA of B95-8 cells and the DNAs of four independent clones of newly obtained EBV-BACs were digested with the indicated restriction enzymes and subjected to Southern blot analysis. A pool of BamHI-digested restriction fragments of AK-BAC was used as a probe. The BamHI-digested bands of B95-8 EBV genome are indicated by alphabets, including Nhet (spanning the TR region), C (spanning the FR region), H (spanning IR2), I (BAC insertion locus), K (spanning IR3), and W (representing IR1). The new bands resulting from the insertion of BAC vector sequence and GFP/hyg^r^ transgenes are indicated by arrowheads. (B) Two independent clones, 189-kb BAC and 174-kb BAC, were digested with NcoI, and the sizes of the bands representing the BamHI W repeats were determined. (C) The genomic DNA of B95-8 cells, and the DNAs of B95.8/F, 189-kb BAC, and 174-kb BAC were double-digested with EcoRI and MluI and analyzed by Southern blot analysis. An ethidium bromide-stained agarose gel picture (left) and a corresponding Southern blot result using the same probe as in Figs. 3B and D (right) are shown.

We employed a similar experimental strategy as previously described [14] for BAC-cloning of the EBV genome. A linear targeting vector, consisting of a BAC vector sequence, a hygromycin resistance gene, a GFP gene, and flanking regions of the EBV genome (Fig. 4A), was stably transfected into the B95-8 cell line. Numbers of cell clones harboring homologously recombined EBV episomes were obtained (Fig. 4B), and homologously recombined BAC clones were recovered from each of the cell clones. We chose four independently obtained BAC clones and examined whether they stably retained the repetitive sequences, including the FR sequence. The newly obtained EBV-BAC clones were digested by BamHI, EcoRI, or NcoI, and the sizes of the restriction fragments were compared with those from genomic DNA of B95-8 cells digested with the same enzymes by Southern blot analysis, again using BamHI-digested restriction fragments of AK-BAC. We readily noticed that, in three (Clones 1 through 3) of the four BAC clones, the lengths of IR1 region, which consists of multiple copies of BamHI W repeats, were identical to that of the parental B95-8 strain EBV (Fig. 5A, NcoI digestion pattern). On the other hand, the length of IR1 region of Clone 4 was apparently shorter (Fig. 5A, NcoI digestion pattern), demonstrating that Clone 4 had decreased copies of BamHI W repeats. Further restriction mapping data revealed that Clones 1 through 3 contained 11 copies of the W repeats while Clone 4 contained six copies of the W repeats (Fig. 3B and data not shown). The entire sizes of BAC Clones 1 through 3 were calculated to be 189-kb, while that of BAC Clone 4 was calculated to be 174-kb. These clones are referred to as “189-kb BAC” and “174-kb BAC” hereafter.

Importantly, the BamHI C fragments of all the obtained BAC clones were identical to those of B95-8 genome (Fig. 5A), making a sharp contrast with the case of AK-BAC (Fig. 3A). The FR sizes of the 174-kb BAC and 189-kb BAC were examined by Southern blot analysis. The result revealed that the FR bands of both 189-kb BAC and 174-kb BAC were identical to the FR band of latently infected B95-8-strain EBV (Fig. 5C). Curiously, we found the FR of a representative BAC clone recovered from 2089 cells [HEK293 cells harboring wild-type EBV-BAC (designated as B95.8/F) [14]] was approximately 300 bp smaller than that of latently infected B95-8 strain EBV (Fig. 3D). We are not aware of whether the particular 2089 cells, which we have maintained in our lab, harbor peculiar B95.8/F clone (with partially deleted FR), or similar deletion is generally occurring in all of the B95.8/F-derived BAC clones.

The bands representing TR, IR2, and IR3 were identical between the latently infected B95-8-strain EBV and all four of the newly obtained EBV-BAC clones (Fig. 5A). The “IR4”, which is located near the right lytic origin of replication, is not present in the B95-8-strain EBV [17], [18], [19] (Fig. 1A and Fig. 4A).

Thus, the overall results indicate that the 189-kb BAC clone retains all of the repetitive sequences of the B95-8-strain EBV genome, while the 174-kb BAC clone does so except for the decreased copy number (six copies) of IR1.

### The FR sequences were stably maintained during progeny virus production and B-cell transformation

We introduced the newly obtained BAC clones (189-kb BAC and 174-kb BAC) into HEK293 cells (Fig. 4B) and examined whether we could produce high-titer recombinant viruses. Multiple GFP-positive cell clones were obtained for both 189-kb BAC and 174-kb BAC, and these cells were examined for their capability to produce infectious viruses. When 174-kb BAC was used for transfection, after screening of GFP-positive cell clones, we obtained several cell clones that were competent for producing highly infectious recombinant viruses (Fig. S3). By contrast, when 189-kb BAC was used for transfection, even after screening of substantial numbers of GFP-positive cell clones, none of the cell clones produce recombinant viruses with high infectious titer for unknown reason.

We therefore proceeded to characterize the recombinant virus of the 174-kb BAC. One representative HEK293-derived cell clones harboring transduced 174-kb BAC clones was chosen for the further analyses. Viral lytic replication was induced by transfection of either BZLF1 expression vector or BZLF1 plus BALF4 (gp110) expression vectors, and culture supernatants containing the recombinant viruses were obtained. Quantitative evaluation of B-cell transformation efficiency of the 174-kb BAC virus revealed that the 50% transforming dose per milliliter (TD_50_/ml) was 10^4.3^ (when induced by BZLF1) and 10^5.5^ (when induced by BZLF1 plus BALF4). Another lot of 174-kb BAC virus induced by BZLF1 plus BALF4 gave TD_50_/ml value of 10^5.3^. On the other hand, the TD_50_/ml value of BZLF1-induced B95-8 virus with a similar EBNA-inducing titer was 10^4.6^. Thus, the 174-kb BAC virus faithfully represents the transforming property of its parental B95-8 virus.

**Figure 6:**
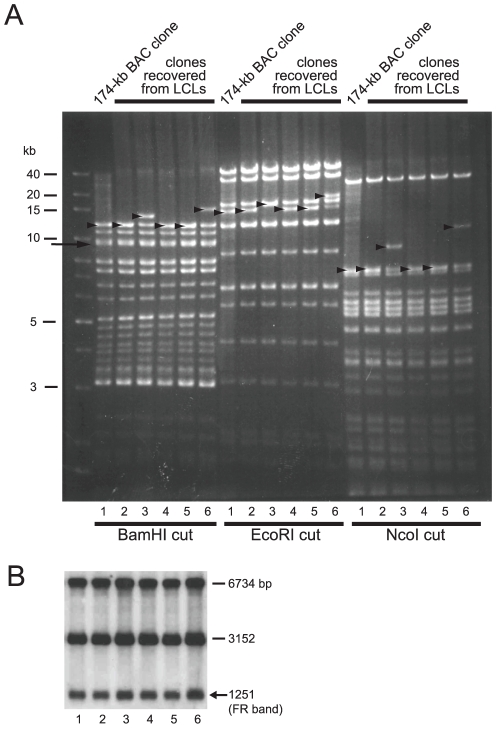
Stable maintenance of the FR of B95-8-derived EBV-BAC clone during virus production and B-cell immortalization. (A) The DNAs of 174-kb BAC (lane 1) and five independent BAC clones that were recovered from the LCLs (lanes 2 to 6) were digested by BamHI, EcoRI, and NcoI, and analyzed by agarose gel electrophoresis. Note that the bands of BamHI C fragments (indicated by an arrow, co-migrating with BamHI B fragments) are constant, whereas the bands representing TR (indicated by arrowheads) are variable. (B) The FR sizes of the same BAC clones as in (A) were analyzed by Southern blot as described in the legend of Fig. 5C.

Multiple GFP-positive LCLs harboring 174-kb BAC were established (Fig. 4B). Episomal fractions were prepared from the LCLs and used to transform DH10B competent cells to recover individual BAC clones. A representative result of restriction enzyme mapping of the recovered BAC clones is shown in Fig. 6A. This result revealed that the recovered BAC clones were identical to the 174-kb BAC clone that was initially used to establish the HEK293-derived virus-producing cells, with the sole exception of the varied lengths of the TR (Fig. 6A). Importantly, the sizes of the BamHI C fragments spanning the FR region were constant among all of the *E. coli*-derived BAC DNAs. A Southern blot analysis revealed that all of the examined BAC clones retained the full-length FR (Fig. 6B), which is in contrast to the case of the Akata-derived BAC clones (Figs. 3C and D).

Thus, the full-length FR of 174-kb BAC was maintained during the processes of stable transfection into HEK293 cells, progeny virus production from HEK293 cells, and B-cell infection and transformation. These results represent the first direct demonstration that the full-length FR sequence of the B95-8 strain of EBV, although it has a highly palindromic nature, can be stably maintained during the life cycle of EBV.

### Comparison of primary sequences of the FR regions of Akata and B95-8 strain EBV

**Figure 7:**
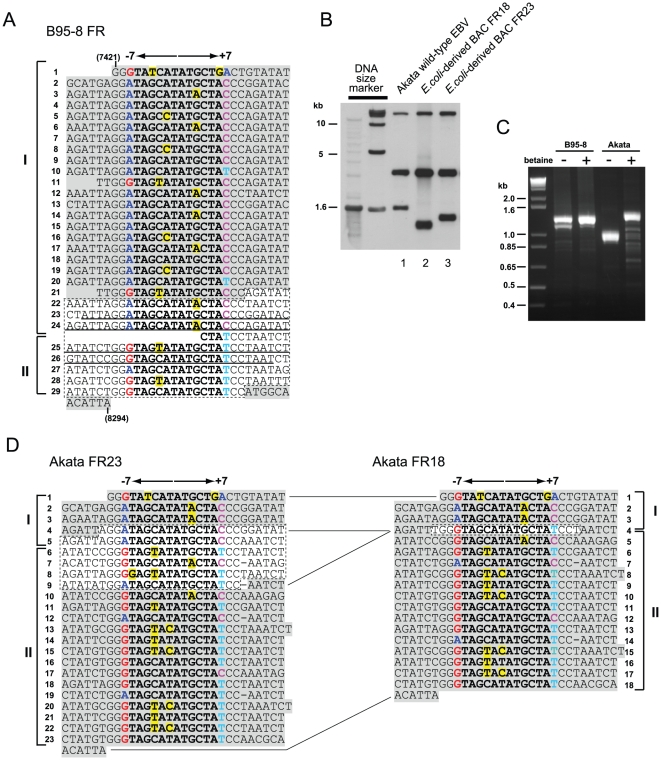
Primary DNA sequences of the B95-8 and the Akata FR. (A) The previously reported B95-8 FR sequence (AJ278309) [5] aligned along palindromic cores of each repeat unit. Nucleotides constituting 12 bp palindromic core of each repeat unit are in bold letters, and those that are mismatched to the consensus motif (TAGCATATGCTA) of the palindromic core are highlighted in yellow. Nucleotides G, A, T, and C at position −7 and +7 are labeled with different colors to emphasize the orientation of each repeat unit. Nucleotides in concordance with the GenBank sequence of the B95-8 strain EBV (V01555) is highlighted in gray, while the 252-bp sequence missing in V01555 is surrounded by dotted lines. Underlined nucleotides represent 128-bp nearly perfect palindrome. Nucleotide numbers in parentheses are as in Fig. 1B. (B) Two Akata-derived BAC clones that were subjected to DNA sequencing of their FR regions were analyzed by Southern blot as in Fig. 3D. (C) B95-8 genomic DNA and Virion DNA of the Akata strain EBV were subjected to PCR analysis, in the absence or the presence of betaine, using a set of primers flanking the FR region. (D) Primary DNA sequences of Akata FR23 (AB644408) and FR18 (AB644409). Bold letters, colored letters, and yellow highlights are as in (A). Identical nucleotides between FR23 and FR18 are highlighted in gray, while discrepancies are surrounded by dotted lines.

Our study so far clearly indicates that the stability of FR sequences, when cloned in BAC vectors, is different between the Akata and the B95-8 strain of EBV. The full length FR of the B95-8 strain EBV consists of 29 copies of 30-bp repeat units [5] (Fig. 7A). On the other hand, based on the size of EcoRI-NcoI fragment in Southern blots (Fig. 7B), we estimate that the FR of the Akata strain EBV consists of 32 copies of 30-bp repeats [11], which is slightly longer that the B95-8 FR. We set up PCR reactions to amplify the B95-8 and the Akata FR by using a set of primers (KA2 and KA3) flanking the FR region [5]. The PCR band representing the full length Akata FR (expected be around 1350 bp) became visible only when a chemical reducing the formation of DNA secondary structures, betaine [20], was included in the PCR reaction (Fi. 7C). The PCR result implicates that the DNA of Akata FR forms a secondary structure *in vitro*.

Unfortunately, our attempt to sequence the PCR product of the full length FR of the Akata strain EBV was not successful. As an alternative approach, we sequenced the FR of two Akata-derived BAC clones with partially deleted FR (Fig. 7B). The obtained sequences revealed that one clone has 23 copies of 30-bp repeats and the other clone has 18 copies of repeats (designated as FR23 and FR18, respectively) (Fig. 7D). The sequences of FR23 and FR18 are identical in their upstream and downstream regions, implicating that internal repeats of the full length Akata FR (consisting of 32 copies of 30-bp repeats) are commonly missing in FR23 and FR18, and that FR18 has suffered larger deletion than FR23. Interestingly, as has been noticed [5], distribution of nucleotides on both sides of 12-bp central palindromic sequence (at −7 and +7 positions) let us identify the orientations of 30-bp repeat units in the B95-8 FR as well as in the Akata FR23. As a result, the FR region can be divided into two regions of repeats which are inverted with respect to each other (regions I and II in Figs. 7A and D). Mfold program [21] predicts that the 252-bp sequence, which is missing in the GenBank sequence of the EBV B95-8 strain (V01555), has a potential to form a DNA secondary structure when it becomes single-stranded (Fig. S4). The same program predicts that DNA sequence spanning repeat no. 4 through 9 of the Akata FR23, when it becomes single-stranded, also can form a secondary structure (Fig. S4). This prediction is compatible with the above PCR result that betaine enabled the PCR amplification of the full length Akata FR. Since it is well known that the DNA sequences constituting secondary structures are susceptible to deletion in *E. coli* [22], this could explain why the Akata strain FR is extremely unstable in *E. coli*-based cloning vectors.

## Discussion

The EBV genome contains multiple repetitive sequences, including IR1 through 4, TR, and FR. Among these repetitive sequences, the FR sequence appears to be the most unstable in *E. coli*-based plasmid vectors due to its palindromic nature [5], [6], [11]. In spite of its critical roles in viral latent infection, the stability of the FR sequence of BAC-cloned EBV has never been examined in detail. We found that the FR of the Akata-strain EBV was not maintained at full-length in the BAC vector (Figs. 3B, C, and D). Importantly, FR instability was observed in two independently-obtained Akata-BAC clones (Figs. 3C and D). Therefore, this is a general problem of the Akata-derived EBV BAC clones. By contrast, the FR of the B95-8 strain could be maintained at full-length in multiple BAC clones (Figs. 5C and 6B).

A previous study demonstrated that the FR sequences of various EBV strains, including B95-8 strain, contain two clusters of inversely-oriented repeats (Fig. 7A) [5]. In case of B95-8, the 252-bp sequence is occasionally lost in *E. coli*-based cloning vectors, most likely due to its formation of a DNA secondary structure when it becomes single-stranded during DNA replication (Fig. S4). In analogy to B95-8 strain, the FR of the Akata strain EBV, when it becomes single-stranded, can probably form a secondary structure at the boundary of the inversely-oriented clusters of repeats. Thus, a possible scenario explaining the instability of FR in EBV BAC clones is that, when the FR region becomes single-stranded in replicating *E. coli-*plasmids, a palindrome-induced deletion occurs by a slipped-mispairing mechanism [22]. We speculate that a very large (spanning up to 400-bp) palindromic structure exists within the Akata strain FR, which results in its unusual instability in *E. coli*-based cloning vectors.

The Akata-derived EBV-BAC clone has been a valuable experimental tool for EBV genome engineering [11], [23], [24], [25]. We argue the obtained results are still valid, as the full length FR was restored in EBV-positive Akata cells in some cases [23], [24], while the partially deleted FR functioned normally after the BAC clones being introduced into P3HR-1 cells to generate mixture viruses [11], [25]. We believe that restoration of the deleted FR (Fig. 3D, from lane 3 to lane 2) occurred via homologous recombination of *E. coli*-derived BAC DNA with helper virus (pre-existing wild-type EBV episome) inside the cells, because such restoration never occurred in the absence of helper virus (data not shown). On the other hand, the full-length FR of the B95-8 strain EBV was stably maintained in the *E. coli*-derived 174-kb BAC DNA, and the DNA was successfully transferred back into cells, in the absence of any helper virus, to produce a recombinant virus harboring the full length FR.

Various critical roles are assigned to the FR sequence, such as stable maintenance of EBV episomes [26], [27], [28] and proper transcriptional regulation of viral latent genes [10]. Therefore, it is tempting to speculate that whether or not the FR is intact could significantly affect the properties of recombinant EBVs. The high B-cell transformation efficiency of the recombinant 174-kb BAC EBV could be due to its retention of the full-length FR, although we have not yet performed genetic analysis to prove the idea. It is still a technical challenge to replacing the full length FR with the deleted FR and subsequently putting the full length FR back to the same place without leaving a trace of any marker gene. Difficulty also resides in getting various virus-producing cells (wild-type, mutant, and revertant) with similar infectious titers, as quite a bit of BAC-transduced HEK293 cells must be screened to obtain those. Clearly, further technical improvement must be accomplished in the future.

We have also noticed that the 174-kb BAC is not completely free from FR deletion. Very minor population with partially deleted FR appeared upon large scale propagation of *E. coli* harboring the 174-kb BAC (data not shown). Furthermore, when we performed recombinogenic engineering of 174-kb BAC in *E. coli*, some of the engineered BAC clones were found to have partially deleted FR (data not shown). We could avoid such a pitfall by regularly checking the integrity of FR during recombinogenic engineering in *E. coli* and by rescuing BAC clones from stably transduced HEK293 cells.

We successfully produced highly infectious recombinant virus derived from the 174-kb BAC clone, but not derived from the 189-kb BAC clone for unknown reason. A previous report demonstrated that the appropriate genome size for being packaged into virions is around 175-kb [29]. Thus, one possibility is that DNA of the 189-kb BAC clones is too big to be efficiently packaged into virions. In case of the 174-kb BAC clone, decreased copies of W repeats (11 to 6 copies) counterbalance the increased size caused by the transgene insertion.

In summary, we found that the FR sequence of the Akata strain of EBV is unstable in BAC vectors. The FR instability can be a limiting factor for current EBV genetics utilizing EBV-BAC clones, and invention of *E. coli* strain that can stably keep all kinds of viral repetitive sequences should be quite beneficial to all area of herpesvirus genetics. Our study also highlights the importance of checking the integrity of viral repetitive sequences during the course of viral genome manipulation.

## Materials and Methods

### Cells

Akata cell line (an EBV-positive Burkitt's lymphoma cell line) [13], B95-8 (a marmoset lymphoblastoid cell line immortalized by EBV) cell line [12], and HEK293 cell line [30] were maintained in RPMI 1640 medium (Sigma-Aldrich Fine Chemicals, St. Louis, Mo.) supplemented with 10% fetal bovine serum.

### BAC-cloning of B95-8-strain EBV genome

A targeting construct for the BAC-cloning of the B95-8-strain EBV genome was generated by using conventional DNA cloning techniques. A green fluorescent protein (GFP) marker gene driven by CAG promoter [derived from pCAGGS [31], a hygromycin phosphotransferase gene (hyg^r^) driven by SV40 early promoter-enhancer, and the BAC vector sequence of pBeloBAC11 were tandemly cloned into pBluescript (Stratagene) vector with PacI recognition sites flanking these transgenes. The pBluescript sequence was then removed by PacI digestion followed by self-ligation. The right homology arm [from NheI site (nt. 143210) to BsmI site (nt. 153213)] and the left homology arm [from ClaI site (nt. 149930) to NheI site (nt. 154909)], both representing the subgenomic regions of the B95-8 strain EBV genome (V01555), were designed according to the previous report [14] with minor modifications. The right and left homology arms were excised from p31 cosmid [32].

The targeting vector (5 µg) was linearized by a single cutting enzyme NheI. B95-8 cells (5×10^6^ cells) were then transfected with the linearized targeting vector via electroporation (Bio-Rad Gene Pulser II; 230 V, 950 µF). Transfected cells were plated at the density of 10^4^ cells per well in 96-well tissue culture plates in medium containing 150 µg of hygromycin (Calbiochem) per ml. Half of the culture medium was replaced with fresh hygromycin-containing medium (100 µg/ml) every 5 days. Drug-resistant cell clones were screened by Southern blotting for the presence of homologously recombined viral DNAs.

EBV episomes were isolated from hygoromycin-resistant cell clones harboring homologously recombined viral DNAs by an alkaline lysis procedure as described previously [16], and one microliter of each episomal preparation was used to transform electrocompetent DH10B cells.

Large quantities of BAC clone DNAs were prepared from each 250 ml bacterial culture by using a Nucleobond BAC100 kit (Macherey-Nagel, Duren, Germany).

### Southern blot analysis

For checking the stability of repetitive sequences (IR1, IR2, and TR) of newly obtained EBV-BAC (B95-8 strain), the genomic DNA of B95-8 cells (2 µg) and aliquots of EBV-BAC DNA minipreparations were digested with BamHI, EcoRI, and NcoI. The digested DNAs were electrophoresed over night in 0.8% agarose gel and then transferred to Hybond N+ membrane. One microgram of BamHI-digested AK-BAC DNA [16] was labeled by AlkPhos Direct kit (GE Healthcare) according to manufacturer's instruction. Hybridization, washing, and signal detection were done according to manufacturer's instruction.

For checking the FR sizes of various EBV-BAC clones, the genomic DNA (1.25 µg each) of EBV-positive cells (either Akata cells or B95-8 cells), and various EBV-BAC clone DNAs (either aliquots of minipreparations or 40 ng each of purified BAC DNAs) were digested by either EcoRI-NcoI (Akata-strain EBV) or EcoRI-MluI (B95-8-strain EBV). The digested DNAs were subjected to 0.8% agarose gel electrophoresis and then transferred to Hybond N+ membrane according to manufacturer's instruction. The PstI fragment of the BamHI C fragment of B95-8 strain EBV or the XcmI-MluI fragment of pCEP4 (Invitrogen) were used as probes (Fig. 1B). Probe labeling and hybridization were performed as described above.

### Recombinant virus production

The HEK293 cells (4×10^5^ cells) were plated in 6-well tissue culture dishes and transfected with a BAC clone DNA (1 µg each, prepared by Nucleobond BAC100 kit) by using lipofectamine 2000 (Invitrogen). At 2 days posttransfection, the transfected cells were re-plated at the density of 4,000 cells per well in 96-well tissue culture plates in medium containing 150 µg of hygromycin per ml. Hygromycin-resistant cell clones with bright GFP fluorescence were grew up, and cell clones that were highly competent for entering lytic replication after BZLF1 transfection were selected.

HEK293 cells carrying recombinant EBV episomes were transfected with either BZLF1 expression vector or BZLF1 plus gp110 expression vectors [33] by using lipofectamine 2000 reagent. Culture supernatants containing recombinant viruses were harvested at 3 days posttransfection and filtered through 0.45 µm-pore-size filter.

### Transformation assay

Blood samples were obtained from healthy adult donors, who gave written informed consent, according to protocols approved by the institutional review board of Aichi Cancer Center, Nagoya, Japan. Peripheral blood mononuclear cells were isolated and infected with serially-diluted (10^−1^ to 10^−5^) culture supernatants containing recombinant EBVs and plated at the density of 1×10^5^ cells per well in 96-well tissue culture plates in medium containing cyclosporine A (500 ng/ml). Half of the culture medium was replaced with fresh medium containing cyclosporine A every 5 days. The number of wells with proliferating cells was counted at 5 weeks postinfection. The established lymphoblastoid cell lines (LCLs) were expanded in the culture medium containing hygromycin (50 µg/ml).

### PCR amplification of the FR region and DNA sequencing

Virion DNA of Akata strain EBV was prepared from the culture supernatant of lytically-replicating Akata cells as previously described [34]. The FR regions was amplified by PCR using a set of primers (KA2 and KA3) [5] and PrimeSTAR (Takara) as a PCR enzyme. When indicated, betaine (final 1 M) was included in the PCR reaction. The PCR parameters were as follows: 98°C for 2 min, 35 cycles at 98°C for 10 s, 60°C for 5 s, and 72°C for 90 s, followed by 72°C for 5 min.

For DNA sequencing, the FR regions of the Akata-derived BAC clones (FR23 and FR18) were amplified by PCR using a set of primers (INT-S and INT-AS) [5] and PrimeSTAR (Takara) as above. The PCR product was purified by QIAquick PCR purification kit (QIAGEN) and subjected to cycle sequencing (FASMAC Co.,Ltd). The DNA sequence of FR23 was verified by CUGA DNA sequencing (Nippon Gene Co., Ltd) as well. Accession numbers for FR23 and FR18 are AB644408 and AB644409, respectively.

## Supporting Information

Figure S1 **Absence of wild-type episome in the re-infected cell clone.** Genomic DNAs of Akata cells harboring wild-type episomes, of those harboring neo^r^ episomes, and of re-infected cell clone harboring AK-BAC episomes were prepared, and they were subjected to a PCR analysis to check the sizes of inserts at BamHI X region. Note that an approximately 11-kb band (arrow) is present in the lane of re-infected cell clone harboring AK-BAC episomes (lane 3), and the same lane is free of 0.85-kb band derived from wild-type episomes (arrowhead). PCR primers and inserted transgenes are schematically illustrated below the panel.
(EPS)

Figure S2 ***E.coli*** **-derived BAC DNA had partially deleted FR.** DNAs of 24 independent AK-BAC-GFP clones rescued from cell clone 12–15 (lanes 1 to 24) were subjected to Southern blot analysis as in Fig. 3D.
(EPS)

Figure S3 **Infectious titers of the 174-kb BAC-derived recombinant virus.** Infectious titers of the recombinant viruses of 174-kb BAC were evaluated by their EGFP-inducing and EBNA-inducing abilities against EBV-negative cell lines. (A) EBV-negative Akata cells were infected with 174-kb BAC virus, and infected cells were analyzed by fluorescence-activated cell sorting using FL1 and FL2 channels at 48 h postinfection. The percentages of GFP-expressing cells were identified by the shift of fluorescence intensity in the FL1 channel. Virus induction was carried out as indicated at top. (B) Infected cells were analyzed by immunofluorescence to detect EBNA expression (bottom). The corresponding differential interference contrast (DIC) images are shown (top).
(EPS)

Figure S4 **Examples of predicted secondary structures of the FR sequences.** The 252-bp sequence missing in the GenBank sequence of the B95-8 strain EBV (V01555) (surrounded by dotted lines in Fig. 7A) and a part of Akata FR23 DNA sequence (surrounded by dotted lines in Fig. 7D) were subjected to MFOLD analysis. The boundaries of 30-bp repeat units are indicated by arrowheads, and numbers represent repeat no. as in Figs. 7A and D.
(EPS)
